# Current trends in psychotherapies and psychosocial interventions for people with dementia: a scoping review of randomized controlled trials

**DOI:** 10.3389/fpsyt.2024.1286475

**Published:** 2024-01-22

**Authors:** Célia Vicente, Sónia Fernandes, Ana Romão, Júlio Belo Fernandes

**Affiliations:** ^1^ Department of Nursing, Hospital Garcia de Orta, Almada, Portugal; ^2^ Nurs Lab, Caparica, Almada, Portugal; ^3^ Egas Moniz Center for Interdisciplinary Research (CiiEM), Egas Moniz School of Health & Science, Caparica, Almada, Portugal

**Keywords:** dementia, Alzheimer disease, cognitive therapies, cognition therapy, rehabilitation

## Abstract

An outcome of dementia is a progressive decline in cognitive function. Implementing psychotherapies and psychosocial interventions is crucial for bolstering cognitive abilities, promoting independence, and elevating the quality of life for individuals with dementia. This review aims to identify current trends in psychotherapies and psychosocial interventions for people with dementia. A Scoping review was developed based on the framework proposed by Arksey and O’Malley. The literature search was conducted on electronic databases, including Scopus, Cochrane Central Register of Controlled Trials, MEDLINE, CINAHL, Nursing & Allied Health Collection, and MedicLatina. Executed in June 2023, the search focused on articles published in English, Portuguese, and Spanish between 2013 and 2023. Through this search, 1409 articles were initially identified. After selecting and analyzing the reports, sixteen trials were included in this review. Eight distinct categories were identified, covering different strategies. These categories run from computerized game-based cognitive training and reminiscence therapy to compensatory and restorative strategies, memory and attention training, calculation training, dual-task training, counseling, and personalized goal attainment. The findings of this scoping review highlight the diverse landscape of psychotherapies and psychosocial interventions for people with dementia.

## 1 Introduction

In the past fifty years, socioeconomic development has been accompanied by significant declines in fertility rates and a simultaneous exponential increase in life expectancy. Consequently, there has been a global phenomenon of population aging (1), with several challenges associated with this aging population (2, 3), including dementia (4).

Dementia is a health challenge characterized by a decline in cognitive function, affecting memory, thinking, orientation, comprehension, calculation, learning capacity, language, and judgment. It is a progressive condition primarily affecting older individuals, leading to significant impairment in daily functioning and quality of life (5, 6). As the global population ages, the prevalence of dementia in Organization for Economic Co-operation and Development countries is estimated to increase from over 21 million to approximately 42 million individuals in 2050 (7).

As the disease progresses, people with dementia depend on a carer to fulfill their basic needs, resulting in an increased caregiver burden (8). In addition, the behavioral and psychological symptoms associated with dementia, such as depression, agitation, and aggression, entail additional difficulties for caregivers and healthcare professionals (8–10).

Caregiving for someone with dementia can be overwhelming, leading to burnout and decreased quality of life (8, 10, 11). The burden of dementia has significant economic implications, with healthcare costs and productivity losses associated with the condition expected to continue to rise (12, 13).

Considering the profound repercussions of dementia, there is an urgent need to create efficient interventions targeting both impairment and participation restriction in individuals suffering from this condition.

The American Psychiatric Association categorizes psychotherapies and psychosocial treatments for dementia into four distinct approaches: (1) behavior-oriented approaches (e.g., behavior therapy), (2) emotion-oriented approaches (e.g., supportive therapy, reminiscence), (3) cognition-oriented approaches (e.g., skills training, reality orientation), and (4) stimulation-oriented approaches (e.g., exercise, art therapy, music therapy, psychomotor therapy) (14).

Although these interventions cannot change the course of the disease, they can support patients to compensate for cognitive deficits and maximize their remaining abilities, addressing both impairment and participation restriction (15 **).**

Combined methods, including both pharmacological and non-pharmacological treatments, have shown significant promise in improving functionality for individuals with dementia. These comprehensive strategies have the potential to slow the progression of the disease, helping individuals maintain their abilities and engage in meaningful activities for a more extended period (16).

Studies by Bahar‐Fuchs et al. (17), Li et al. (18), and Orgeta et al. (19) have all demonstrated the effectiveness of cognitive interventions in enhancing memory, attention, executive function, and problem-solving skills, resulting in overall improvements in cognitive performance and quality of life.

Additionally, these interventions can provide valuable strategies for caregivers to manage challenging behaviors and enhance communication with individuals living with dementia (20, 21).

Identifying psychotherapies and psychosocial interventions for people with dementia is of utmost importance. It allows health professionals to stay updated with the latest advancements and ensure that interventions are evidence-based. In addition, health professionals and researchers can build upon existing knowledge and refine their approaches to better address the specific needs of people with dementia. Therefore, this review aims to identify current trends in psychotherapies and psychosocial interventions for individuals with dementia.

## 2 Methods

This scoping review was drawn based on the framework outlined by Arksey and O’Malley (22) and subsequently expanded upon by Levac et al. (23). In addition, we adhered to the checklist provided by the Preferred Reporting Items for Systematic Reviews and Meta-Analyses extension for Scoping Reviews (PRISMA-ScR) (24).

### 2.1 Search methods

The research question that guides the review is: What are the current trends in psychotherapies and psychosocial interventions for individuals with dementia?

We developed a search strategy to uncover pertinent literature based on the ‘Population–Concept–Context (PCC)’ mnemonic. The PCC served as the basis for determining the criteria for inclusion and exclusion (**Table 1**).

**Table 1:** Eligibility criteria.

Parameter	Inclusion criteria	Exclusion criteria
Population	• Individuals diagnosed with dementia; • Individuals with mild cognitive impairment or greater; • Adults ≥ 18 years old.	• Other health conditions besides dementia; • Individuals without cognitive impairment or with subjective cognitive impairment; • Participants < 18 years old.
Concept	• Studies that explore psychotherapies and psychosocial interventions. There are no limitations on the intervention type, provided it aimed to enhance or prevent cognitive dysfunction.	• Studies that do not address psychotherapies and psychosocial interventions.
Context	• Studies conducted in rehabilitation settings (e.g., home, acute, post-acute, and long-term care).	
Study design	• Randomized controlled trials	
Language	• English, Portuguese, and Spanish.	

Publications from 2013 to 2023 were identified in a comprehensive electronic search via Scopus, Cochrane Central Register of Controlled Trials, MEDLINE, CINAHL, Nursing & Allied Health Collection, and MedicLatina.

We incorporated Medical Subject Heading terms. The following combinations of search terms were used: [(Dementia OR Alzheimer disease) AND (Cognitive training OR Cognitive therapies OR Cognition therapy) AND (Rehabilitation)].

The final search was done on June 16, 2023.

### 2.2 Study selection

Duplicated identification was identified, individually reviewed, and removed by CV using Rayyan - AI-Powered Tool for Systematic Literature Reviews. The same researcher performed the screening of manuscript titles to determine potential relevance. CV and SF performed the abstract screening independently, after which they reviewed full-text articles concerning the eligibility criteria. Any discrepancies were settled via discussion and consensus between the reviewers and the involvement of a senior reviewer (JBF) if required.

### 2.3 Data extraction

A data extraction form was developed and used by CV and JBF to collect relevant data from the included studies. For each review, the following data were extracted: study characteristics (e.g., authors, publication year, study design, aim) and intervention details (e.g., type of intervention, duration, frequency, and length).

### 2.4 Data synthesis and analysis

The extracted data were analyzed to identify the current trends in psychotherapies and psychosocial interventions for individuals with dementia. The characteristics and key findings of the included studies were summarized. Based on the guidelines proposed by Braun et al. (25), we conducted a data-driven thematic analysis to identify interventions used in people with dementia. Patterns and consistencies in the reported outcomes across the studies were explored. No statistical analysis or meta-analyses were possible due to significant heterogeneity observed among the included studies, including variations in intervention types, timing of sessions, and settings. Therefore, the findings were extracted and reported systematically.

## 3 Results

**Figure 1:**
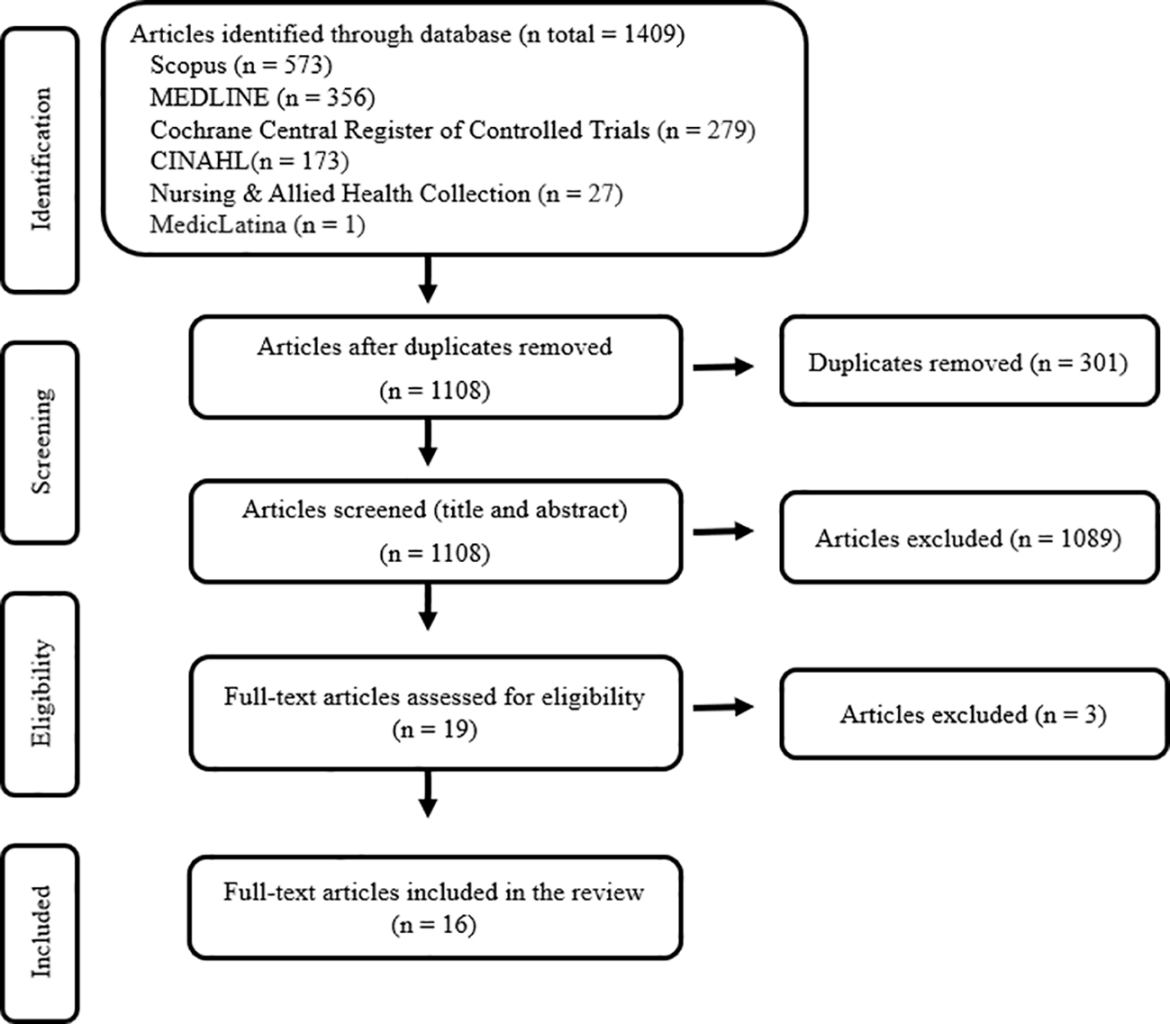
PRISMA flow chart for study selection.

After a comprehensive search in multiple databases, we initially identified 1409 studies. After the duplicate removal, 1108 studies remained, of which 1089 were excluded based on titles and abstracts. Ultimately, 19 publications were read in full text, and 16 studies that met the eligibility criteria were included in this review. **Figure 1** illustrates the selection process.

The 16 randomized controlled trials were published between 2013 and 2023. Out of the 16 trials, there were six conducted in Europe (26–31), two in Japan (32, 33), two in China (34, 35), two in Republic of Korea (36, 37), one in the United States (38), one in Australia (39), one in Mexico (40), and one in Turkey (41).

A list of included randomized controlled trials is shown in **Table 2**.

**Table 2:** Data extraction and synthesis.

Author/Year/Title/Country	Study Design/Aim/Participants	Time/Frequency/Length	Intervention	Results
Greenaway et al. (38) The memory support system for mild cognitive impairment: randomized trial of a cognitive rehabilitation intervention. United States	Randomized controlled trial To examine the efficacy of a calendar/notebook rehabilitation intervention, the memory support system, for individuals with amnestic mild cognitive impairment. 40 participants with mild cognitive impairments and their program partners.	60 minutes Twice a week Six weeks	• Compensatory and Restorative strategies	Significant enhancements were observed in functional ability and memory self-efficacy among participants in the intervention group. The intervention group consistently maintained a significantly better functional ability improvement than the control group throughout the 8-week follow-up period. Care partners in the intervention group exhibited improved mood levels at the 8-week and 6-month follow-ups, whereas control care partners reported an increase in caregiver burden by the 6-month follow-up.
Amieva et al. (26) Group and individual cognitive therapies in Alzheimer’s disease: the ETNA3 randomized trial. France	Randomized controlled trial To compare the effect of cognitive training, reminiscence therapy, and an individualized cognitive rehabilitation program in Alzheimer’s disease to usual care. 653 participants with mild to moderate cognitive impairment.	90 minutes Weekly sessions Three months and maintenance sessions every six weeks for the next 21 months	• Reminiscence therapy • Calculation training	No impact on the primary efficacy measure was evidenced. For the two group interventions (i.e., cognitive training and reminiscence), none of the secondary outcomes differed from usual care. The larger effect was observed with individualized cognitive rehabilitation, demonstrating significantly reduced functional disability and a delayed six-month institutionalization at the two-year mark.
Nakamura et al. (32) The Group Reminiscence Approach Can Increase Self-Awareness of Memory Deficits and Evoke a Life Review in People With Mild Cognitive Impairment: The Kurihara Project Data. Japan	Randomized controlled trial To investigate the effects of group reminiscence approach and reality orientation for participants with mild cognitive impairment using the patient report outcome. 94 participants mild cognitive impairment.	60 minutes Weekly sessions Twelve weeks	• Reminiscence therapy	The intervention group engaged in reminiscence through a life review, reflecting on their memory issues. Notably, there was no confusion regarding the chronological order of events in their autobiographical memories.
Han et al. (36) Efficacy of the Ubiquitous Spaced Retrieval-based Memory Advancement and Rehabilitation Training (USMART) program among patients with mild cognitive impairment: a randomized controlled crossover trial. Republic of Korea	Randomized controlled trial To investigate the efficacy of USMART in patients with mild cognitive impairment. 43 participants with mild cognitive impairment	30 minutes Twice a week Four weeks	• Computerized game-based cognitive training	The intervention group exhibited greater improvements in the Word List Recall Test compared to the usual care group. No significant differences were observed in other primary or secondary measures between the two groups.
Regan et al. (39) MAXCOG-Maximizing Cognition: A Randomized Controlled Trial of the Efficacy of Goal-Oriented Cognitive Rehabilitation for People with Mild Cognitive Impairment and Early Alzheimer Disease. Australia	Randomized controlled trial To review the efficacy of a home-based four-session individualised face to face cognitive rehabilitation (MAXCOG) intervention for clients with mild cognitive impairment mild cognitive impairment or early dementia and their close supporters. 40 participants with mild cognitive impairment and 40 supporters.	60 minutes Four a week Four weeks	• Personalized Goal Attainment	The intervention group demonstrated significantly higher performance and satisfaction with primary goals on the Canadian Occupational Performance Measure post-intervention compared to the control group.
Hindle et al. (27) Goal-orientated cognitive rehabilitation for dementias associated with Parkinson’s disease-A pilot randomised controlled trial. United Kingdom	Randomized controlled trial To examine the appropriateness and feasibility of cognitive rehabilitation for people with dementias associated with Parkinson’s in a pilot randomised controlled study. 29 participants with Parkinson’s disease dementia or dementia with Lewy bodies and 26 carer participants.	60 minutes Weekly sessions Eight weeks	• Personalized Goal Attainment • Compensatory and Restorative strategies	At two months, the group intervention showed superior outcomes compared to the control group in self‐rated goal attainment and self‐rated satisfaction with goal attainment. This superiority persisted at six months for self‐rated goal attainment. Caregivers in the intervention group reported higher quality of life, improved health status, and lower stress levels than those in the control group.
Shimada et al. (33) Effects of Combined Physical and Cognitive Exercises on Cognition and Mobility in Patients With Mild Cognitive Impairment: A Randomized Clinical Trial. Japan	Randomized controlled trial To compare the cognitive and mobility effects of a 40-week program of combined cognitive and physical activity with those of a health education program. 308 participants with mild cognitive impairment.	90 minutes Weekly sessions 40 weeks	• Dual-task training • Counseling	Compared with the control group, the combined activity group showed significantly greater scores on the Mini-Mental State Examination and Wechsler Memory Scale-Revised Logical Memory II, significant improvements in mobility and the nonmemory domains, and reduced left medial temporal lobe atrophy in amnestic mild cognitive impairment.
Clare et al. (28) Individual goal-oriented cognitive rehabilitation to improve everyday functioning for people with early-stage dementia: A multicentre randomised controlled trial (the GREAT trial). United Kingdom	Randomized controlled trial To determine whether individual goal‐oriented cognitive rehabilitation (CR) improves everyday functioning for people with mild‐to‐moderate dementia. 474 participants with mild to moderate cognitive impairment.	60 minutes Weekly sessions Three months followed by maintenance sessions over the subsequent six months.	• Counseling • Personalized Goal Attainment	At three months, participant-rated goal attainment had statistically significant positive effects. These effects were maintained at nine months—the observed gains related to goals directly targeted in the therapy. There were no significant differences in secondary outcomes.
Law et al. (34) Effects of functional tasks exercise on cognitive functions of older adults with mild cognitive impairment: a randomized controlled pilot trial. China	Randomized controlled trial To validate the effects of functional tasks exercise on cognitive functions and functional status in older adults with mild cognitive impairment. 59 participants with mild cognitive impairment.	60 minutes Twelve sessions Eight weeks	• Computerized game-based cognitive training	The intervention group demonstrated greater memory and caregiver burden improvements compared to the exercise and cognitive training groups.
Lemke et al. (29) Transferability and Sustainability of Motor-Cognitive Dual-Task Training in Patients with Dementia Germany	Randomized controlled trial To examine transfer effects and the sustainability of a specific dual-task training in patients with dementia. 105 participants with mild to moderate dementia.	90 minutes Twice a week Ten weeks	• Dual-task training	Compared to the control group, the intervention group exhibited significant improvements in dual-task performances, encompassing motor, cognitive, and combined motor-cognitive dual-task. Three months after the cessation of training, dual-task performance in the intervention group remained elevated for most outcomes.
Kim (37) The effects of dementia partner programs using telephone on cognitive and neuropsychiatric symptoms in elderly persons with mild cognitive impairment: A randomized controlled trial Republic of Korea	Randomized controlled trial To examine the effects of dementia partner programs on cognition, depression, and quality of life in elderly persons with mild cognitive impairment residing in a community. 23 participants with mild cognitive impairment.	Five to ten minutes Four a week Ten weeks	• Memory and attention training • Calculation training • Counseling	The intervention group demonstrated significant improvements in the cognitive and self-esteem domains, whereas the control group exhibited statistically significant enhancements in the quality of life and self-esteem domains. Analysis of the variations by group indicated that the scores of all items showed positive effects in both groups, while cognition and the quality of life showed statistically significant differences in the experimental group.
Peng et al. (35) The Efficacy of Cognitive Training for Elderly Chinese Individuals with Mild Cognitive Impairment. China	Randomized controlled trial To evaluate the cognitive function in elderly people using the Montreal Cognitive Assessment, to identify the relationship between cognitive function and different characteristics, and to evaluate the efficacy of the intervention after six months of cognitive training. 2886 participants with mild cognitive impairment, dementia, or neurological diseases.	90 minutes Every two weeks Six months	• Memory and attention training • Calculation training	The total score on the Montreal Cognitive Assessment for the intervention group increased after six months of cognitive training, while the control group experienced a decrease. Statistical analysis revealed a highly significant interaction effect between time and training on the total Montreal Cognitive Assessment score.
Juárez-Cedillo et al. (40) Randomized Controlled Trial of Multi-Component Cognitive Stimulation Therapy (SADEM) in Community-Dwelling Demented Adults. Mexico	Randomized controlled trial To evaluate the effectiveness of a multicomponent cognitive stimulation therapy (SADEM) on cognitive and behavioral function and daily life activities in patients with mild stage dementia. 67 participants with mild dementia	90 minutes Twice a week Twelve months	• Memory and attention training • Counseling	The findings indicated statistically significant differences, demonstrating improved cognitive outcomes and the Dementia Index post-intervention. No progression of the disease was observed after the study.
Manenti et al. (30) Effectiveness of an Innovative Cognitive Treatment and Telerehabilitation on Subjects With Mild Cognitive Impairment: A Multicenter, Randomized, Active-Controlled Study. Italy	Randomized controlled trial To evaluate the efficacy of the face-to-face cognitive virtual reality rehabilitation system and to compare it to that of face-to face cognitive treatment as usual for individuals with mild cognitive impairment. 49 participants with mild cognitive impairment.	60 minutes Twelve sessions Four weeks	• Computerized game-based cognitive training	Following the completion of face-to-face virtual reality rehabilitation system treatment, memory, language, and visuo-constructional abilities were enhanced compared to traditional face-to-face treatment.
Torpil et al. (41) The Effectiveness of a Virtual Reality-Based Intervention on Cognitive Functions in Older Adults with Mild Cognitive Impairment: A Single-Blind, Randomized Controlled Trial Turkey	Randomized controlled trial To evaluate the effectiveness of a virtual reality-based rehabilitation program on cognitive functions in mild cognitive impairment. 61 participants with mild cognitive impairment	45 minutes Twice a week Twelve weeks	• Computerized game-based cognitive training	The intervention group exhibited significantly greater improvements in orientation, visual-spatial perception, visuomotor organization, thinking operation, and attention/concentration functions than the control group.
van Balkom et al. (31) Effect of eight-week online cognitive training in Parkinson’s disease: A double-blind, randomized, controlled trial. Netherlands	Randomized controlled trial To assess the efficacy of adaptive, computerized cognitive training on cognitive function in Parkinson’s disease. 136 participants with mild cognitive impairment.	45 minutes Three times a week Eight weeks	• Computerized game-based cognitive training	Analyses showed no group difference on the Tower of London accuracy corrected for baseline performance. At follow-up, no group differences were found.

Data analysis unveiled a range of psychotherapies and psychosocial interventions for people with dementia. These rehabilitation interventions have been categorized into eight distinct categories.

### 3.1 Computerized game-based cognitive training

Several studies have explored the effectiveness of computerized game-based cognitive training in enhancing cognitive function in individuals with varying degrees of cognitive impairment (30, 31, 34, 36, 41). While these studies share the approach of using computerized games to stimulate cognitive abilities, they differ in terms of methodology, intervention strategies, and outcomes.

A study by Han et al. (36) introduced a spaced retrieval-based memory training program using an iPad tablet. Participants were tasked with memorizing and immediately recalling words displayed on the screen. This self-administered approach showed promise in mitigating cognitive decline in individuals with mild cognitive impairment. Similarly, Law et al. (34) employed computerized games within a supervised group setting led by an occupational therapist. The training encompassed multiple cognitive functions and demonstrated improvements over eight weeks.

Manenti et al. (30) took a more immersive approach and utilized a virtual reality rehabilitation system to train skills such as memory, spatial orientation, and executive functions. In contrast, Torpil et al. (41) explored using commercially available games with the Microsoft Kinect for virtual reality-based intervention, highlighting the potential benefits even from entertainment-based games.

Van Balkom et al. (31) introduced an adaptive online cognitive training program based on the ‘Braingymmer’ platform. It targeted attention, processing speed, and executive functions, adjusting its difficulty according to each participant’s performance. Meanwhile, Lemke et al. (29) employed dual-task training through computerized game-based motor-cognitive training on an interactive balance platform alongside a motor learning exercise program focusing on compensatory sit-to-stand maneuvers. Each study provided valuable insights into the benefits of computerized game-based cognitive training, with variations in platforms, delivery methods, and emphasis on specific cognitive skills.

### 3.2 Reminiscence therapy

Nakamura et al. (32) and Amieva et al. (26) employed group-based reminiscence therapy to enhance cognitive engagement and well-being. Nakamura et al. (32) facilitated discussions among participants centered on daily or seasonal events from their youth, fostering a collective exploration of past experiences. This approach was complemented by integrating visual aids, including pictures and relevant items, to deepen sensory connections and enrich the reminiscence therapy experience.

Similarly, Amieva et al. adopted a group-based approach but introduced a structured program for reminiscence therapy. Their tailored program focused on diverse personal themes, spanning schooldays, birthdays, weddings, working life, and holidays. By delving into these distinct life themes, participants were guided to evoke memories associated with specific events, potentially enhancing cognitive engagement through an organized and targeted strategy.

### 3.3 Compensatory and restorative strategies

Greenaway et al. (38) introduced a compact Memory Support System featuring a two-page per day calendar and a note-taking system tailored to conveniently fit within a man’s breast pocket or a woman’s purse. This system was devised to offer a practical means of organizing and retaining daily information, enhancing memory recall and daily functioning.

Conversely, Hindle et al. (27) took a broader approach by incorporating strategies to manage cognitive difficulties, practical situations, and anxiety symptoms. These strategies encompassed the utilization of compensatory tools such as calendars, diaries, and reminders. Additionally, they incorporated restorative techniques like mnemonic devices and spaced retrieval to bolster the retention of new information and improve recall.

### 3.4 Memory and attention training

Peng et al. (35) explored Memory and Attention Training using diverse techniques. Their strategy encompassed a Seven-piece board recovery training to challenge and enhance memory recall. They also employed activities like picture-reading memory exercises and phrase recitation. Additionally, they targeted attention development through color reaction training and Schulte Grid exercises.

Taking a unique perspective on Memory Training, Kim (37) introduced an innovative dementia partner program. This one-on-one approach was facilitated via telephone and focused on engaging participants in discussions about news, sharing daily routines, and planning for upcoming days or weeks.

In addition to these studies, Juárez-Cedillo et al. (40) contributed a multi-component cognitive stimulation program encompassing cognitive aspects like paying attention and maintaining orientation.

### 3.5 Calculation training

Peng et al. (35) employed a Calculation Training strategy involving two straightforward questions and one simple application question for calculation within each intervention session.

Similarly, Amieva et al. (26) developed a program designed to engage various cognitive functions and activities of daily life. Their approach included Calculation Training through tasks such as money counting to improve domestic financial handling.

Kim (37) introduced a distinctive dementia partner program that utilized telephone interactions. This program featured tailored cognition items, including calculation checking for specific weekdays, counting numbers in reverse, related word listing games, and pair word telling.

### 3.6 Dual-task training

Shimada et al. (33) utilized Dual-Task Training by combining aerobic exercise, muscle strength training, and postural balance retraining with cognitive tasks.

As previously mentioned, Lemke et al. (29) also incorporated Dual-Task Training. However, their approach involved computerized game-based motor-cognitive training on an interactive balance platform.

### 3.7 Counseling

In the context of counseling interventions, several studies have employed distinct strategies to address various aspects of health and well-being.

Shimada et al. (33) implemented a counseling approach in which instructors educated participants on aging, nutrition, oral care, frailty, and urinary incontinence. The participants also received informative pamphlets on these subjects throughout the study period.

Kim (37) utilized telephone counseling, covering a range of items, including physical self-monitoring, stress intervention, health promotion, mental self-monitoring, nutrition and diet, social competence, challenges in the community, and monitoring physical and mental changes. This comprehensive approach aimed to provide holistic guidance to individuals.

Juárez-Cedillo et al. (40) employed counseling to foster personal interaction, boost self-esteem, and enhance personal security. Their approach aimed to address psychological and emotional well-being, further enriching the scope of counseling interventions.

Clare et al. (28) employed counseling to assist participants in defining and achieving up to three rehabilitation goals through a problem-solving approach. They also addressed emotional and motivational challenges by applying emotion regulation and behavioral activation strategies as necessary.

### 3.8 Personalized goal attainment

Hindle et al. (27) introduced a Goal-Oriented Cognitive Rehabilitation program. This intervention embraced evidence-based techniques to guide participants in pursuing agreed-upon goals. The program was structured to align with each participant’s objectives, promoting a targeted and personalized approach to cognitive enhancement.

Clare et al. (28) similarly assisted patients in defining rehabilitation goals using a problem-solving approach. Regan et al. (39) offered a personalized intervention called MAXCOG, which honed in on individually significant goals. This approach involved learning names at social events, enhancing medication adherence, and boosting memory for specific activities. Both interventions were tailored to everyone, recognizing and addressing their cognitive needs and challenges.

## 4 Discussion

The review evaluated various interventions targeting cognitive enhancement and rehabilitation across multiple categories. A substantial body of literature has examined the effects of computerized game-based cognitive training interventions (30, 31, 34, 36, 41). These studies contribute to understanding the versatility of computerized game-based cognitive training interventions. The consistent findings across these studies suggest that technology-driven interventions can engage participants while targeting a range of cognitive functions. The successful integration of technology into cognitive rehabilitation strategies opens avenues for more personalized, engaging, and adaptable interventions tailored to diverse populations, from mild cognitive impairment to stroke survivors.

Nakamura et al. (32) and Amieva et al. (26) provided insight into the use of reminiscence therapy. Group-based reminiscence therapy, supported by visual aids and tailored themes, emerged as a valuable approach for engaging individuals in recalling and sharing past experiences. This therapy promotes memory recall and offers potential benefits for emotional well-being and social interaction.

Studies by Peng et al. (35), Juárez-Cedillo et al. (40), and Kim (37) highlighted the versatility of memory training interventions. Approaches varied from seven-piece board recovery training, tailored tasks at different difficulty levels, to unique dementia partner programs via telephone. These interventions emphasize the potential to enhance memory through various techniques, ranging from challenging exercises to personalized interactions.

Lemke et al. (29) and Shimada et al. (33) have explored integrating motor and cognitive therapy in dual-task training interventions. This method combines physical exercises with cognitive tasks, showing a synergistic effect that enhances motor-cognitive integration and overall performance. Lemke et al. (29) study offers a fresh perspective by incorporating technology into dual-task training. While combining motor and cognitive tasks is not new, their use of interactive balance platforms and computerized game-based exercises introduces innovation. This technological integration enhances the approach’s complexity and engagement, boosting its potential for motor-cognitive integration and functional outcomes.

The overarching benefit of combined motor and cognitive therapy lies in its potential to reflect the complex cognitive demands of everyday life (42–44). This approach aligns with emerging theories of brain plasticity, suggesting that the brain’s adaptability can be harnessed through integrated interventions (45, 46). As cognitive and motor functions intertwine in real-life scenarios, targeting both domains simultaneously in therapy may lead to more efficient and comprehensive improvements in overall functionality.

The review also highlighted compensatory and restorative strategies. Compensatory strategies, supported by Greenaway et al. (38) and Hindle et al. (27), offer practical solutions by using external aids to navigate cognitive challenges. Memory support systems, calendars, and reminders assist in organizing thoughts and managing tasks. In contrast, as emphasized by Hindle et al. (27), restorative strategies aim to enhance cognitive function through targeted training and interventions. Cognitive exercises, problem-solving techniques, and memory training work to regain lost cognitive abilities and enhance cognitive reserves.

Training executive functions in individuals with dementia through interventions like Memory and Attention Training and Calculation Training is profoundly significant, improving their quality of life and potentially slowing symptom progression. These functions include planning, decision-making, organization, problem-solving, and emotional control. Impairments in these areas are characteristic of dementia and significantly affect daily life and independence (47, 48).

Beyond direct enhancements in memory, attention, and logical thinking, these training methods offer broader benefits, including increased social engagement, heightened self-esteem, and reduced emotional stress. However, customization to individual needs, considering disease stage, personal preferences, and cognitive capacities, is crucial. Hindle et al. (27), Clare et al. (28), and Regan et al. (39) underscored tailored interventions focusing on individual goals, aligning cognitive rehabilitation with personally meaningful objectives to enhance motivation and outcomes.

The review’s findings highlight diverse interventions, emphasizing the absence of a universal approach to enhance cognitive function and participation restriction. In the broader context, a comprehensive strategy is crucial to meet the diverse needs of those with cognitive impairments and participation restriction.

No single intervention is a panacea. Instead, cognitive rehabilitation emerges from the synergy of various interventions, addressing specific cognitive challenges. By embracing various strategies, a holistic program can be designed to accommodate each patient’s unique strengths and weaknesses.

Ultimately, a well-rounded rehabilitation program for people with dementia acknowledges the multifaceted nature of cognitive impairments. By offering personalized interventions, professionals can optimize benefits for individuals, empowering them to enhance cognitive abilities, regain independence, and elevate overall quality of life.

### 4.1 Challenges and considerations

While psychotherapies and psychosocial interventions show promise, challenges must be addressed to ensure their effectiveness and widespread implementation. One challenge is the diverse nature of dementia, requiring tailored interventions to suit individual cognitive profiles and symptom severity (3, 49, 50).

Although many studies demonstrate short-term positive effects (15, 17, 51), maintaining cognitive improvements over extended periods remains a concern, necessitating longer follow-up studies. In addition, translating cognitive gains from interventions to real-world situations poses another challenge, requiring ecologically valid training programs (52).

Advancing technology brings new tools for cognitive rehabilitation, potentially revolutionizing the field with engaging, personalized, and accessible interventions (53). Implementation, however, demands multifaceted considerations.

For users, accessibility and usability are paramount, especially for individuals with varying tech familiarity or cognitive impairments. User-friendly, intuitive technology tailored to cognitive abilities preserves user dignity and autonomy, enhancing engagement.

Healthcare professionals play a crucial role, requiring effective technology training. Access to progress tracking ensures effective implementation, necessitating seamless technology integration into healthcare systems.

Economically, evaluating the cost-effectiveness of technology-based interventions is vital. Balancing upfront costs with long-term benefits like improved cognition and enhanced quality of life guides resource allocation.

Ensuring affordability and equity for individuals, families, and healthcare systems is vital, making technology-based interventions accessible regardless of socioeconomic status.

### 4.2 Strengths and limitations

This review’s key strength lies in its focus on experimental studies, allowing for a precise exploration of current trends in psychotherapies and psychosocial interventions for individuals with dementia. This review offers valuable insights into promising cognitive training and rehabilitation approaches by explicitly pinpointing interventions subjected to rigorous experimental scrutiny. The comprehensive overview of these interventions facilitates a deep comprehension of the techniques and methodologies employed, simplifying their replication in other settings.

Nevertheless, certain limitations do exist in this research. Not all studies provide exhaustive descriptions of interventions, potentially hindering their replication by other professionals. By exclusively focusing on experimental studies, there is a risk of overlooking valuable insights from non-experimental studies, which could enrich the overall comprehension of cognitive training and rehabilitation interventions. Additionally, limiting the search to Scopus and EBSCOhost research platforms might have excluded pertinent studies from alternative sources. Furthermore, specific criteria, such as excluding non-English, Portuguese, and Spanish papers, could introduce biases by omitting relevant literature. Finally, by following the framework outlined by Arksey and O’Malley, this scoping review does not include the assessment of study quality as a component of the scoping process. Recognizing these limitations is crucial to maintaining a balanced interpretation of the findings and encouraging further research that transcends this review’s scope.

## 5 Conclusion

This review facilitated the synthesis of evidence on psychotherapies and psychosocial interventions for individuals with dementia and captured current trends encompassing various approaches, ranging from computerized game-based cognitive training and reminiscence therapy to compensatory and restorative strategies, memory and attention training, calculation training, dual-task training, counseling, and personalized goal attainment.

Dementia poses a significant global health challenge with far-reaching consequences for individuals, families, and society. Cognitive training and rehabilitation interventions are vital in enhancing cognitive functioning, independence, and quality of life for individuals with dementia. Continued research, innovation, and collaboration across disciplines are necessary to develop and refine interventions that can make a meaningful difference in the lives of individuals with dementia.

## Data availability statement

The data that support the findings of this study are available from the corresponding author upon reasonable request. Requests to access these datasets should be directed to juliobelo01@gmail.com.

## Author contributions

CV: Conceptualization, Data curation, Formal analysis, Investigation, Methodology, Project administration, Supervision, Writing – original draft, Writing – review & editing. SF: Formal analysis, Investigation, Methodology, Writing – original draft, Writing – review & editing. AR: Investigation, Methodology, Writing – original draft, Writing – review & editing. JF: Conceptualization, Data curation, Formal analysis, Investigation, Methodology, Supervision, Writing – original draft, Writing – review & editing.
